# Books and Phamplets Received

**Published:** 1887-02

**Authors:** 


BOOKS AND PAMPHLETS RECEIVED.—We acknowledge with thanks the receipt of the following publications:

Transactions Iowa State Medical Association, 1886.
Report State Board of Health, New Hampshire, 1886.
Transactions of the Medical Society of Pennsylvania, 1886.
" Mississippi State Medical Association, 1886.
" New Hampshire Medical Society, 1886.
" Tennessee Medical Society, 1886.
" Louisiana Medical Society, 1886.
" Missouri Medical Society, 1886.
" North Carolina Medical Society, 1886.
Medical Communications, Massachusetts Medical Society, 1886.
Transactions Medical Association of Georgia, 1886.
Eighth Annual Report Illinois State Board of Health, 1885.
An Epitome of the Newer Materia Medica, from Messrs. Parke, Davis & Co., Detroit.

All of which we hope to be able to review at some future time.

